# Effect of Different Packaging Methods on Protein Oxidation and Degradation of Grouper (*Epinephelus coioides*) During Refrigerated Storage

**DOI:** 10.3390/foods8080325

**Published:** 2019-08-07

**Authors:** Xicai Zhang, Wenbo Huang, Jing Xie

**Affiliations:** 1 College of Food Science & Technology, Shanghai Ocean University, Shanghai 201306, China; 2 Shanghai Engineering Research Center of Aquatic Product Processing and Preservation, Shanghai 201306, China; 3 Shanghai Professional Technology Service Platform on Cold Chain Equipment Performance and Energy Saving Evaluation, Shanghai 201306, China; 4 National Experimental Teaching Demonstration Center for Food Science and Engineering, Shanghai Ocean University, Shanghai 201306, China; 5 Jingchu University of Technology, Jingmen 448000, China

**Keywords:** grouper, refrigerated storage, packaging methods, protein oxidation, protein degradation

## Abstract

This study investigates the effect of different packaging methods—namely, air packaging (AP), vacuum packaging (VP), and modified atmosphere packaging (MAP)—on the protein oxidation and degradation of grouper (*Epinephelus coioides*) fillets during refrigerated storage. The carbonyl group, myofibril fragmentation index, free amino acids, FTIR of myofibrillar proteins, and total protein SDS-PAGE were determined. The results showed that the protein oxidation degree of the fillets gradually increased as the storage time increased. The FTIR results indicated that the secondary structure transformed from an α-helix to an irregular curl. SDS-PAGE confirmed the degradation of the myosin heavy chain, and that myosin gradually occurred during refrigerated storage. Meanwhile, protein oxidation and degradation were highly correlated. Protein degradation was accelerated by protein oxidation in myofibrils, which included the increase of protein surface hydrophobicity and changes of the secondary structure. In fact, the protein oxidation and degradation of the grouper fillets were effectively inhibited by MAP and VP during refrigerated storage, and MAP (30% N_2_ and 70% CO_2_) had the best results.

## 1. Introduction

Grouper (*Epinephelus coioides*), which belongs to the order Perciformes and the family Serranidae, is a warm-water, offshore demersal fish that is referred to as “marine chicken” because of its considerable similarity in taste to chicken meat. With the development of artificial breeding and breeding technology, grouper has become an important economic fish along the coast of China [1]. Furthermore, the living standards of residents have been significantly enhanced as the economy has developed. Due to the quickening pace of life of the younger generation, fresh fish fillets are becoming the main marketing form of fish products [2,3]. However, grouper can easily decompose, because of its abundant nutrition, high water content, and excellent protease activity. Protein is one of the most important nutrients in aquatic products. During cold storage, the changing forms of protein mainly include protein oxidation and degradation. Carbonyl compounds formed by protein oxidation can change the cell structure of the myofibrillar protein, affecting the hydrophobicity index of fish protein [4,5]. Soon after fish die, the protein initially breaks down into many intermediates with the hydrolysis of endogenous protease [6,7]. In the later stages, the enzymes produced by microbial reproduction lead to protein degradation, resulting in spoilage of the fish. Therefore, the change of biochemical characteristics of proteins in aquatic products is an important reason for the deterioration of such products’ quality during cold storage [8]. Refrigerated fish fillets need to be packaged to ensure their freshness and to meet the needs of different customers. Presently, the main packaging methods of grouper are air packaging (AP), vacuum packaging (VP), and modified atmosphere packaging (MAP). AP and MAP have been considered to be effective ways of preserving food, due to the excellent isolation of oxygen and food [9,10]. MAP, due to the bacteriostatic effect of CO_2_ inducing anaerobic conditions into the packaging environment, can effectively reduce apparent changes of aquatic products [11]. In our previous study, it was found that a high concentration of CO_2_ in MAP (30% N_2_ and 70% CO_2_) could prolong the shelf life of cold-stored grouper. Currently, research on the effects of VP and MAP on the quality of aquatic products mainly focuses on the quality attributes, shelf-life assessment, changes of proteins in aquatic products during storage, and the denaturation of proteins [12,13,14]. Also, trichloroacetic acid (TCA)-soluble peptides, the myofibril fragmentation index (MFI), free amino acids (FAAs), SDS-PAGE, and FTIR of myofibrils are usually evaluated during storage.

However, the relationship between protein oxidation and degradation is controversial. On the one hand, the hydrophobicity caused by protein oxidation increases protease recognition and the subsequent oxidation of protein degradation [15]. On the other hand, the polymer caused by protein oxidation between protein crosslinking could affect the further degradation of proteins, and is a poor substrate of protease [16]—something which has not been studied in research on grouper. It is not clear if MAP or VP can isolate oxygen as an effective method of food preservation and slow down the progress of protein oxidation in grouper. Therefore, the purpose of this study is to investigate the relationship between protein oxidation and degradation, and to compare the preservation effects of protein oxidation and degradation among AP, MAP, and VP refrigerated storage on grouper fillets.

## 2. Materials and Methods

### 2.1. Sampling

Fresh grouper (weight: 500 ± 50 g; length: 35 ± 5 cm) were purchased from the wholesale aquatic market of Luchao Port (Shanghai, China) and immediately transported to the laboratory in crushed ice within 0.5 h.

Based on the cleaning process, the head, bone, and skin of the grouper were removed and drained under refrigerated temperatures. Afterwards, the dorsal part of the fish fillets were cut to 2 × 2 × 2 cm and packaged in polyethylene bags (low-density polyethylene; relative density of 0.917–0.924, 30 × 20 cm). The samples were respectively packaged in AP (meaning they were exposed to air), VP, and MAP (with 30% N_2_ and 70% CO_2_). All the fillets samples were refrigerated at 4.0 ± 0.5 °C. Relative indexes were evaluated regularly at 0, 3, 6, 9, 12, 15, and 18 days. The AP group was terminated at the 12th day due to the deterioration of the grouper fillets.

### 2.2. Extraction of Myofibrillar Protein

The extraction of myofibrillar protein was carried out according to the method of Ogawa et al. [17]. Briefly, 2 g fillets were weighed and washed by a fivefold volume of Tris-HCl buffer (pH 7.0, 10 mmol/L). Then, a fivefold volume of KCl-Tris buffer was added to the abovementioned solutions, followed by homogenization in an ice bath for 90 s (12,000 rpm), with a brief pause in the middle of the homogenization process to prevent overheating. The homogenate was centrifuged three times at 5000× *g* for 10 min. Subsequently, a fivefold volume of 10 mmol/L Tris-HCl buffer (0.6 mol/L NaCl, pH 7.0) was added and centrifuged repeatedly for 10 min at 5000× *g*. The supernatant was a myofibrillar protein extract, which was stored at −80 °C in a refrigerator for further use.

Protein concentration was determined by the method of Abbey et al. [18]. Standard curves were prepared by BSA (Bull Serum Albumin). Protein solution (0.05 mL) was added to 3 mL of Bradford reagent, mixed, and kept still for 10 min. The OD (optical density) value was measured by a spectrophotometer at 595 nm. At the same time, the following protein concentrations were determined by the same method.

### 2.3. Carbonyl Content

The carbonyl content was determined by following the procedures mentioned by Oliver et al. [19]. The myofibrillar protein extract was adjusted to a concentration of 5 mg/mL with phosphate buffer solution (pH 7.0), and incubated in 1 mL 0.01 mol/L 2, 4-dinitrophenylhydrazine solution at 37 °C for 30 min. Then, 3 mL of 20% trichloroacetic acid was added and centrifuged at 8500× *g* for 5 min. The supernatant was removed and the precipitate was washed six times with an ethyl acetate and ethanol mixture solution (1:1, *v*/*v*). Finally, the precipitate was dissolved in 5 mL of guanidine hydrochloride solution (6 mol/L) and incubated for 15 min under a 37 °C water bath, which was centrifuged for 10 min at 8500× *g*. Finally, the absorbance of the supernatant was measured at 370 nm. The carbonyl content was expressed as nmol carbonyl/mg protein.

### 2.4. Surface Hydrophobicity

Surface hydrophobicity was measured as described by Chelh et al. [20]. The abovementioned extracted myofibrillar protein was adjusted to 1 mg/mL with phosphate buffer solution (pH 7.0). Two hundred milliliters of bromophenol blue (1 mg/mL) were mixed with 1 mL of protein solution, followed by constant oscillation for 15 min to react sufficiently. As for the control group, the same procedure was implemented using phosphate solution to replace the extracted myofibrillar protein. Then, centrifugation was done at 4 °C and 2000× *g* for 15 min. The supernatant was diluted 10 times, the absorbance of which was measured at 595 nm. Surface hydrophobicity was expressed by the following formula: bromophenol blue/mg protein = 200 μg × (OD control − OD sample)/OD control.

### 2.5. Total Sulfhydryl and Disulfide Bond Content

Total sulfhydryl and disulfide bond content was evaluated according to the method of Benjakul et al. [21]. One milliliter of myofibrillar protein solution (4 mg/mL) was added to 9 mL 0.2 mol/L Tris-HCl buffer (pH 6.8, 8 mol/L urea, 2% SDS, and 10 mmol/L EDTA (Ethylene Diamine Tetraacetic Acid)). Then, 0.4 mL 5,5-dithio-bis (2-nitrobenzoic acid) (0.1%) was added to the resulting mixture and incubated for 25 min at 40 °C. The absorbance was measured at 412 nm. At the same time, 0.6 mol/L KCl as a blank was also subjected to this step. The extinction coefficient of 13,600 mol^−1^ cm^−1^ was used to calculate the total -SH group content. The content of the disulfide bond was calculated according to the method described by Thannhauser et al. [22].

### 2.6. Ca^2+^ ATPase Activity

The Ca^2+^ ATPase activity was determined by the method described by Benjakul et al. [23]. The content of inorganic phosphate in the supernatant was determined by the method of Thanonkaew et al. [24]. The activity of Ca^2+^ ATPase was defined as the milliliter (nmol/mg protein) of inorganic phosphate produced by 1 mg of protein in 1 min.

### 2.7. Trichloroacetic Acid-Soluble Peptide Content

According to the method of Sriket et al. [25], a 3 g sample was added to a 27 mL TCA solution (5%), which was homogenized by a high-speed tissue homogenizer for 1 min at 12,000 rpm and then placed in an ice bath for 1 h. Repeated centrifugation was carried out at 4 °C and 5000× *g* for 5 min. The result was expressed as umol tyrosine/g muscle.

### 2.8. Myofibril Fragmentation Index

The method of Culler et al. was used to evaluate the MFI [26]. Briefly, the concentration of protein solution was adjusted to 0.5 mg/mL; then, the absorbance was measured at 540 nm, and the MFI value was equal to the OD value multiplied by 200.

### 2.9. Free Amino Acid Content

Samples of free amino acid extracts were prepared according to the method of Yu et al. [27]. Mobile phase 1 of an automatic amino acid analyzer (L-8800, Hitachi Co. Ltd., Tokyo, Japan) consisted of the buffer of sodium citrate and citric acid; the pH of the mixed buffers were 3.2, 3.3, 4.0, and 4.9, respectively. Mobile phase 2 was prepared by 4% ninhydrin (*v*/*v*). The test parameters were as follows: column (4.6 × 150 mm, 7 μm); column temperature (50 °C); channels 1 and 2 flow rates (0.4 mL/min and 0.35 mL/min, respectively).

### 2.10. FTIR Measure

The grouper fillets were powdered with KBr after being freeze-dried (MINFAST04, TIANLI Executive and Administration Management, Beijing, China); then, the mixed sample was pressed into flakes. An FTIR (Nicolet iS5, Thermo Scientific Inc, Waltham, MA, USA) spectrometer was used for the measurements. Infrared spectra were recorded with 32 scans in the 400–4000 cm^−1^ range with a resolution of 4 cm^−1^; also, the operating environment was set at 25 °C. The recorded spectra were analyzed by Omnic professional software (Omnic professional, v 9.2, Thermo Nicole Inc., Waltham, MA, USA), and Gaussian fitting was used to analyze the second-derivative spectrum in the range of 1600–1700 cm^−1^ by PeakFit software (v 412, Systat Software Inc., San Jose, CA, USA).

### 2.11. SDS-PAGE

First, 3 g of minced samples and 30 mL of 50 g/L SDS solution were homogenized at 85 °C. Then, the mixture was subjected to heat preservation for 1 h after high-speed homogenization for 5 min (12,000 r/min), and the supernatant was taken after 5000× *g* for 20 min [28]. SDS-PAGE was performed at a 4–20% gradient, and a real-band, three-color, high-range protein marker purchased from Sangon Biotech (Sangon Biotech Co., Ltd., Shanghai, China) was adopted. A sensitive protein fast staining kit was used for staining, and the decolorized gel was scanned by a gel image scanning system after electrophoresis (GelDoc XR, Bio-Rad Inc., Hercules, CA, USA). Background subtraction, band matching, and optical density calculation were analyzed by Quantity One software (Quantity One 4.0, Bio-Rad Inc., USA).

### 2.12. Statistical Analysis

Three replicates were used for all samples with three parallel tests. SPSS 8.0 (SPSS, Chicago, IL, USA) was used for statistical analysis. Univariate ANOVA was used to determine the statistical differences in different treatment groups. Duncan’s multiple range test was used to determine the significant difference between the averages (*p* < 0.05). The data were expressed as the mean and standard deviation (SD), and Origin 8.5 (OriginLab, Northampton, MA, USA) was used for illustration.

## 3. Results and Discussion

### 3.1. Changes in Total Sulfhydryl and Disulfide Bonds

**Figure 1:**
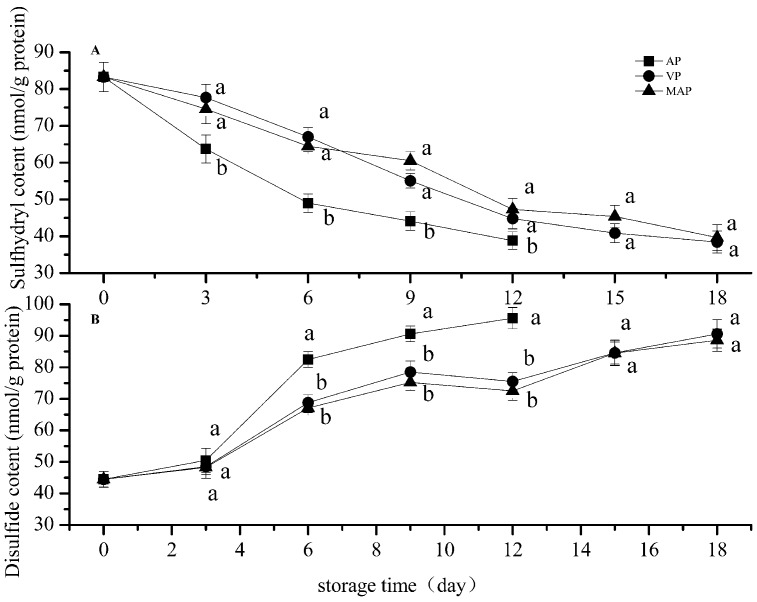
Changes in sulfhydryl (**top**) and disulfide (**bottom**) bond contents of grouper myofibrillar protein (AP: air packaging; VP: vacuum packaging; MAP: modified atmosphere packaging). Different lower-case letters in different groups from same day indicate a significant difference (*p* < 0.05).

Figure 1 shows the changes of sulfhydryl and disulfide bonds in each group. As shown in Figure 1, the content of the total sulfhydryl groups in each treatment group presented a downward trend with the extension of storage time, which is similar to trends found in other studies on grouper (He et al., 2018). The sulfhydryl content of the AP group decreased to 49.05 nmol/mg of protein on the sixth day, which was significantly different compared with the VP and MAP groups. In contrast, the differences between the VP and MAP groups were not significant. The sulfydryl content in the VP group was slightly lower than that in the MAP group after nine days.

The sulfhydryl group of myofibrils can be oxidized by reactive oxygen species (ROS) that produce disulfide bonds, sulfonic acid, and other oxidation products during refrigerated storage [29]. Protein oxidation can be reflected by the loss of the sulfhydryl group [30], which contains a disulfide bond; this is a common oxidation product that can gradually increase with the oxidation of the sulfhydryl group. The disulfide bond content in each group increased gradually with the oxidation of the sulfhydryl group (Figure 1), and the content of the AP group increased significantly, from 44.5 to 82.5 nmol/mg protein. It is worth noting that the disulfide bond contents of the AP and MAP groups decreased slightly starting from the ninth day, which may have been caused by the degeneration of myofibril.

As shown by the two-way analysis of variance, the storage time and packaging methods significantly affected the total sulfhydryl and disulfide bonds of the grouper fillets (*p* < 0.01). There was a significant difference between the VP and AP groups, and between the AP and MAP groups (*p* < 0.01); however, there was no significant difference between the VP and MAP groups (*p* > 0.05).

### 3.2. Changes in Carbonyl Content and Hydrophobicity

**Figure 2:**
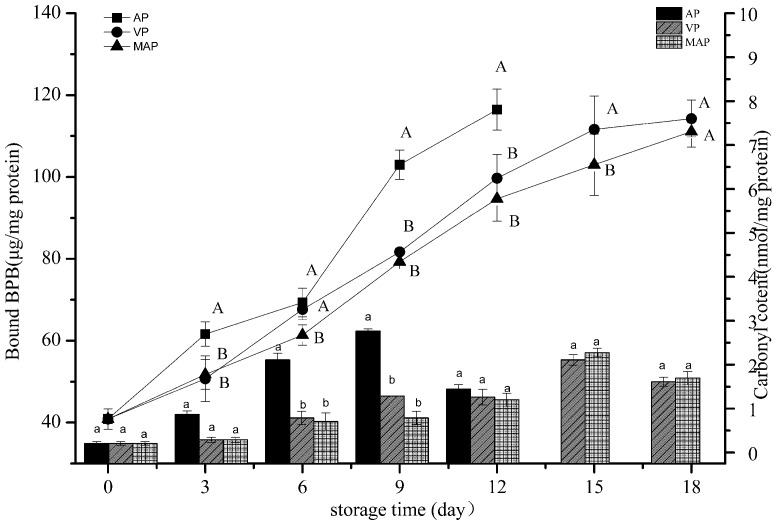
Changes in carbonyl content (bar graph) and protein surface hydrophobicity (expressed as Bound BPB, line graph) of grouper myofibrillar protein. (AP: air packaging; VP: vacuum packaging; MAP: modified atmosphere packaging; BPB: bromophenol blue).

Protein carbonylation is one of the most important changes in the oxidation of muscle proteins, especially the extensive modifications caused by the oxidation of myofibrils. In particular, from the formation of carbonyl compounds [31], changes in the content of carbonyl compounds are usually used to represent the oxidation rate of proteins. In Figure 2, it can be seen that the change of carbonyl content in each group increased with the cold storage time. The initial carbonyl content of the fresh fish was increased 10 times in the AP group on day 6. The two-way analysis of variance revealed that protein carbonylation was significantly affected by storage time and packaging methods (*p* < 0.05).

The specific binding of bromophenol blue with myofibrillar is considered to be a simple and reliable method for the determination of surface hydrophobicity. The hydrophobicity of the protein surface can be determined by the degree to which bromophenol blue binds specifically to myofibrillar [20]. Due to the conformational changes induced by hydroxyl radicals, oxidized myofibrillar protein undergoes extensive exposure of hydrophobic groups [32]. This phenomenon was confirmed in our experiment. The degree to which bromophenol blue bound specifically to myofibrillar became serious in all three groups, which showed no significant differences in the first six days (*p* > 0.05). In the AP group, the content reached 102.98 μg, which was slightly higher than those of the other two groups. However, the VP group was slightly higher than the MAP group on day 15. The change of the surface hydrophobicity of the protein may have been caused by the entry of nonpolar amino acid molecules into hydrophobic clusters [33]. The expansion or rearrangement of protein molecules could lead to a change of the secondary and tertiary structures of the proteins. VP and MAP significantly delayed the oxidation of protein in grouper meat, similar to what has been found in other fish studies [34]. The two-way analysis of variance showed that the storage time and packaging methods significantly affected the carbonyl content and hydrophobicity of the grouper fillets (*p* < 0.01). There was a significant difference between the VP and AP groups, and between the AP and MAP groups (*p* < 0.01); however, there was no significant difference between the VP and MAP groups (*p* > 0.05).

### 3.3. Changes in Ca^2+^ ATPase Activity

Ca^2+^ ATPase mainly concentrates in the globular heads of myosin. The hydrophobic interactions, hydration of polar residues, and hydrogen bonds influence the stability of the three-dimensional structure of the protein. Since the three-dimensional structure of the protein determines the physiological activities of the protein itself, the activities of the protein may be lost or changed because of changes in the microstructure. The Ca^2+^ ATPase activity of actomyosin can be used as an important indicator for assessing the degree of protein denaturation, as it can indirectly reflect the integrity of myofibrillar protein [35].

**Figure 3:**
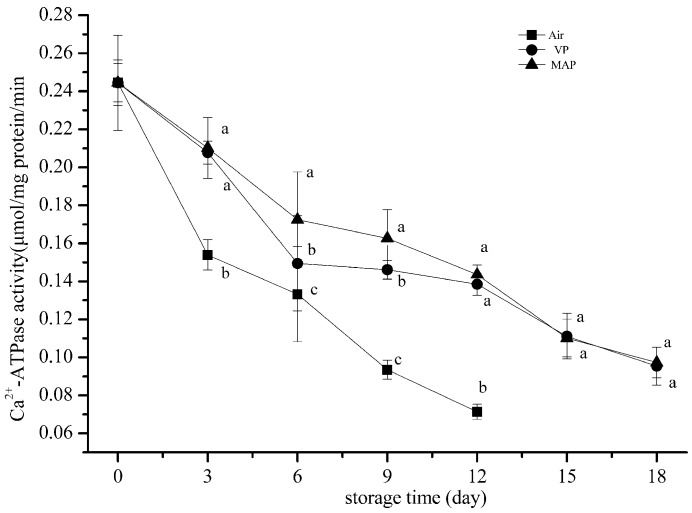
Changes in Ca^2+^ ATPase activity of grouper myofibrillar protein. (AP: air packaging; VP: vacuum packaging; MAP: modified atmosphere packaging).

Figure 3 shows that the Ca^2+^ ATPase activity of the grouper fillets respectively decreased in all samples. The two-way analysis of variance showed that storage time and packaging methods significantly affected the Ca^2+^ ATPase content of the grouper fillets (*p* < 0.05). On the 12th day, the content of the Ca^2+^ ATPase activity in the AP group decreased about 75.16%. There was a significant difference between the VP and AP groups and between the AP and MAP groups (*p* < 0.01), but there was no significant difference between the VP and MAP groups (*p* > 0.05). However, the VP and MAP groups were about 45.83%, which was consistent with the existing reports of other aquatic products [36]. It has been reported that the sulfhydryl group is abundant in the center of Ca^2+^ ATPase [21]. In our experiment, we found that the activity of Ca^2+^ ATPase was closely related to the sulfhydryl group. The correlation coefficients of the AP, VP, and MAP groups were 0.983, 0.946, and 0.984, respectively, and the Ca^2+^ ATPase activity was highly correlated with the change of the sulfhydryl group content. It was speculated that the oxidation of the sulfhydryl group in the active center resulted in a decrease of Ca^2+^ ATPase activity.

### 3.4. Trichloroacetic Acid-Soluble Peptide Analysis

**Figure 4:**
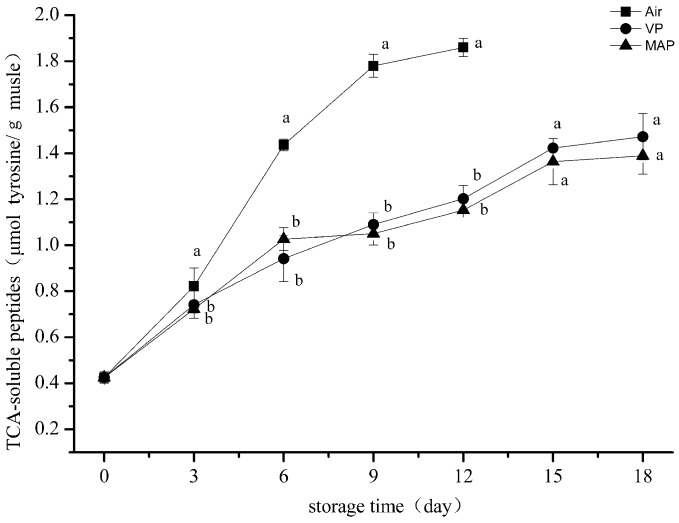
Changes in trichloroacetic acid (TCA)-soluble peptides of grouper myofibrillar protein (AP: air packaging; VP: vacuum packaging; MAP: modified atmosphere packaging).

TCA-soluble peptides can reflect the degree of protein degradation. As shown in Figure 4, the TCA-soluble peptide content in all groups was obviously elevated during the entire storage period. The results showed that proteolysis occurred in succession and was similar to the trend reported by Yu et al. for grass carp fillets under refrigerated storage [37]. The initial number of TCA-soluble peptides of the grouper fillets was 0.42 μmol tyrosine/g sample, while the value reached 1.44 μmol tyrosine/g sample after six days storage for the AP group, which had the largest rate of increase among the three groups. The two-way analysis of variance revealed that the storage time and packaging methods significantly affected the TCA-soluble peptides of the grouper fillets (*p* < 0.01). The increase of TCA-soluble peptides might have initially been due to the activity of the endogenous enzyme [38]. Then, protein degradation was accelerated under the combined action of endogenous enzymes and microorganisms. The final TCA-soluble peptide content of the VP and MAP groups was significantly lower than that of the AP group (*p* < 0.05). The content of TCA-soluble peptides of the MAP group was lower than that of the VP group, but there was no significant difference between the two groups (*p* < 0.05), indicating the effective inhibition of proteolysis by VP and MAP, which might have been due to the growth of microorganisms and inhibition by the anoxic environment in the VP and MAP groups.

### 3.5. Myofibril Fragmentation Index Analysis

**Figure 5:**
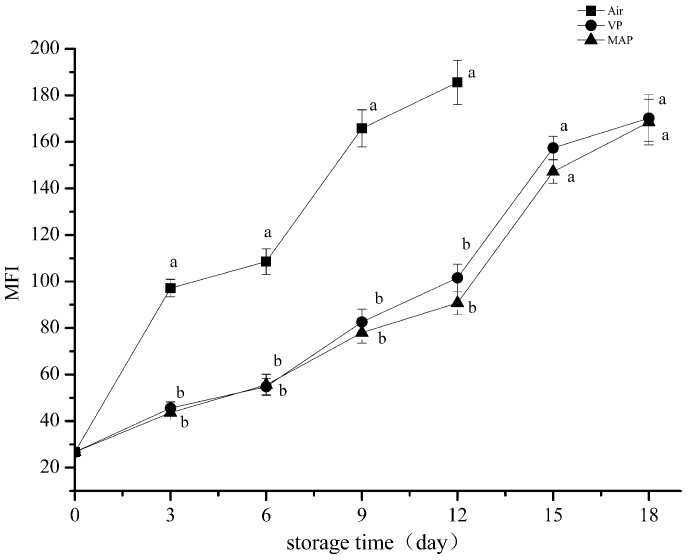
Changes in the myofibril fragmentation index (MFI) values of grouper myofibrillar protein. (AP: air packaging; VP: vacuum packaging; MAP: modified atmosphere packaging).

As shown in Figure 5, the MFI maintained increasing trends among all groups. However, compared with the other two groups, the AP group rose significantly, with 97 mg/100 g being reached on day 3 (*p* < 0.05). No obvious change of the MFI in the VP and MAP groups was discovered at the corresponding time. The two-way analysis of variance revealed that storage time and packaging methods significantly affected the MFI of the grouper fillets (*p* < 0.01). There was no significant difference between the VP and MAP groups on the 12th day, but the MFI of the AP group was very significantly higher (*p* < 0.01) than those of the VP and MAP groups. This indicates that the internal integrity of myofibrillar in the AP group was the most destructive during refrigeration storage, while the destruction of the internal structure of myofibril was delayed by MAP and VP. Myofibril fragmentation refers to the phenomenon of myofibril breaking into shorter segments near the Z disk or Z line. The MFI was calculated from the percentage of myofibrils that were 1–4 sarcomeres long, which was mainly due to degradation of the connectin and actin of sarcomeric I. The MFI reflects the structural integrity of the myofibril during refrigerated storage [39].

### 3.6. Free Amino Acid Analysis

The concentrations of FAAs in grouper fillets on the first, sixth, and twelfth days of refrigerated storage are shown in Table 1. The degradation of protein was the main reason for the variation of FAAs [37], so the degree of protein degradation could be reflected by the changes of the FAA content. A similar trend was also reported by Shi et al. for grass carp [40]. Table 1 shows that the content of FAA rapidly increased with storage time in the AP group, which might have been due to the degradation of protein caused by protein oxidation. However, the content of histidine in the grouper fillets was far below that found in grass carp, as reported by Yu et al., during refrigerated storage, which might be ascribed to the muscle composition of grouper. Aspartic acid, glutamic acid, and glycine are the main flavor contributors in the fillets, and the change of glycine was the most obvious among all groups. Glycine in the AP group increased from 91 mg/100 g on day 0 to 153 mg/100 g on day 6, then it dropped to 78 mg/100 g on day 12. The corresponding contents in the VP and MAP groups were 88 and 108 mg/100 g, respectively. The total FAA contents in the AP group were significantly higher (*p* < 0.05) compared with the other groups on day 6, and this phenomenon indicates that the degree of protein degradation was the greatest in the AP group. The number of FAAs was closely related to the storage time (*p* < 0.05), and the packaging method had no significant effect on it, as shown by the two-way analysis of variance (*p* > 0.05). The final FAA content was reduced among all groups, which when compared to day 6, might have been due to the degradation of enzymes caused by microbial growth [41].

### 3.7. FTIR Analysis

**Figure 6:**
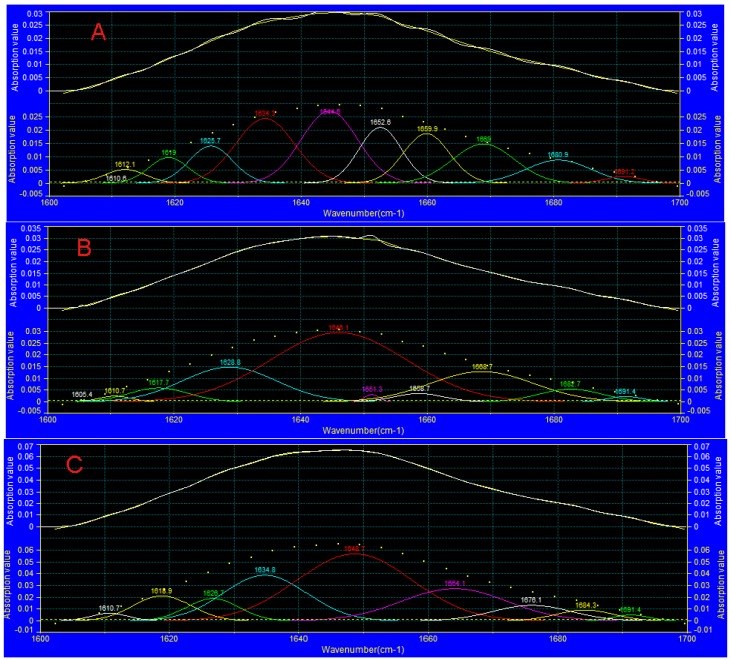

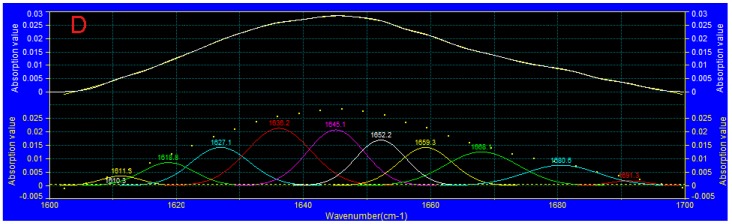
The second-order, second-derivative, mid-infrared spectra by PeakFit. (**A**: 0 days; **B**: AP group on the 12th day; **C**: VP group on the 12th day; **D**: MAP group on the 12th day).

The secondary structure of the protein was composed of an α-helix, a β-sheet, a β-turn, and a random coil. The function of a protein and its biochemical properties change with the variation of the secondary structure, which is due to the oxidation of the protein. As one of the main methods of studying the secondary structure of proteins, FTIR can be used to analyze changes in protein structure and the spatial distribution of proteins [42]. The amide I band of a protein (from 1600 to 1700 cm^−1^ of mid-infrared spectroscopy) can reveal a wealth of information about the constituents of the secondary structure in proteins. The peaks at the wavenumbers of 1600–1640, 1640–1650, 1650–1660, and 1660–1700 cm^−1^ are for the β-sheet, random coil, α-helix, and β-turn, respectively [43]. As shown in Figure 6, the second-order, second-derivative, mid-infrared spectra of the Gaussian fitting drawn by PeakFit (PeakFit v 412, Systat Software Inc., USA) was used to analyze the protein secondary structure changes of each group at days 0 and 12.

Figure 6 shows that the peaks of each packaging group had a certain weakening on the 12th day. The change from the range of 1650–1660 cm^−1^ was significant compared with that of day 0, indicating that the levels of the random coil had increased. As shown in Figure 6A–D, the absorption peak of the spectrum shifted to the high-wavenumber area. The reason for this phenomenon was reported as the hydrogen bond of the protein structure being destroyed during refrigerated storage [44]. Since the spectrum diagram of the MAP group on the 12th day was most similar to that on day 0, compared to the other treatment groups, it indicates that the destruction of the secondary structure of the protein was inhibited in refrigerated grouper fillets in the MAP group.

**Figure 7:**
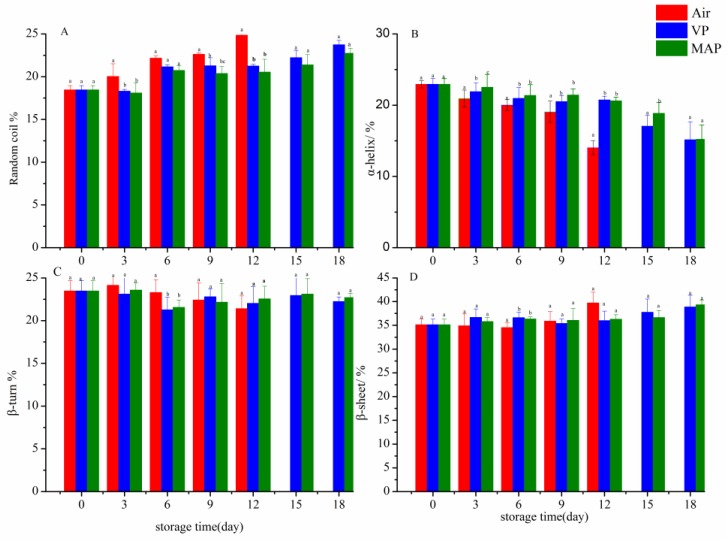
Changes in secondary structure contents of grouper protein during refrigerated storage (**A**: the change in the random coil content under different treatments, **B**: the change in the α-helix content under different treatments, **C**: the change in the β-turn content under different treatments, **D**: the change in the β-sheet content under different treatments). (Air: air packaging; VP: vacuum packaging; MAP: modified atmosphere packaging).

The change in the secondary structure for all groups is shown in Figure 7. The random coil increased, and the α-helix content decreased during refrigerated storage, which might have been due to the breakage of the hydrogen bonds. Further, the surface hydrophobicity increased, and a disordered state gradually became present in the protein. The random coil increased by 34.8% on the 12th day compared to day 0 in the AP group, which was significantly higher than that of the VP and MAP groups (*p* > 0.05). The results showed that the protein structure was partly inhibited by VP and MAP. The β-sheet of each group also increased to a certain extent (*p* > 0.05), due to the change of the peptide chain folding structure caused by the gradual formation of sulfhydryl oxidation and disulfide bonds.

### 3.8. SDS-PAGE Analysis

**Figure 8:**
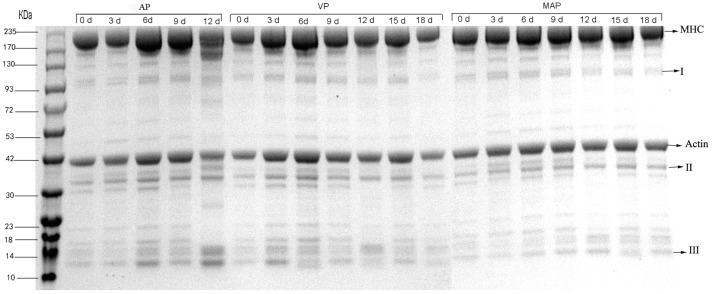
Effects of three packaging methods (AP, VP, and MAP) on total protein degradation of grouper samples during refrigerated storage at 4 °C. (AP: air packaging; VP: vacuum packaging; MAP: modified atmosphere packaging).

The total protein electrophoresis results of the different treatment groups are shown in Figure 8. The results of the band optical density analysis using Quantity One 4.0 showed that the content of the myosin heavy chain (MHC) and actin increased between 0 to 6 days in different groups. Then, different degrees of degradation appeared in different groups after six days. At day 12, the intensity of the MHC in the AP, VP, and MAP groups was 355, 555, and 643, respectively (*p* < 0.05). The MHC, which is very easily oxidized, is crosslinked by disulfide and non-disulfide covalent bonds which contribute to the formation of high-molecular-weight polymers and aggregates [45]. The degradation of MHC was inhibited significantly by VP and MAP. The degradation of protein was probably due to MHC oxidation, and oxidative damage of proteins may have also led to protein degradation and the crosslinking and aggregation of actin, which corresponds to the results of Lu et al. [46]. The actomyosin (42 kDa) band gradually became shallow over time. The change ranges of the VP and MAP groups were significantly smaller than that of the AP group, the main reason for which being the hydrolysis of cathepsin L; the change range of the MAP group was the smallest, indicating that the activity of cathepsin L in the MAP group was inhibited. Band III (13 kDa) of the AP group at day 12 was significantly different than the other two groups, and was highly correlated with reduced actin (correlation coefficient of 0.89), which we concluded was due to the degradation product of actin.

It has not been reported in previous works that all of the intensities of band II (34 kDa) in the three groups showed an increasing trend in the first 12 days. It can be concluded that the intensity of band II, which was speculated to be an indicator protein of grouper freshness before the 12th day, had a highly linear relationship with storage time (*R*^2^ = 0.97), as analyzed by Quantity One 4.0. However, in the VP and MAP groups, the intensity of band II decreased after 12 days. The possible reason for this is that the protein belongs to a water-soluble protein, and the rate of drip loss increases at the later stage of storage [47]. The concentration of protein decreased with water loss, due to the type of protein belonging to water-soluble proteins; such changes can also be observed in bands I and III.

This study showed that the increase of protein oxidation was reflected by the content and distribution of carbonyl and sulfhydryl content changes. Table 2 shows that the carbonyl and sulfhydryl contents of protein oxidation indexes in each group were highly correlated with protein degradation, TCA-soluble peptides, and MFI. This indicates that protein oxidation promotes the degradation process during refrigerated storage of grouper fillets. Compared with AP, VP and MAP exhibited significant positive effects with regard to reducing protein oxidation. However, the MAP group showed better effectiveness than the VP group, which may have been due to the higher residual oxygen and oxygen transmission rate in VP. When vacuum pumping cannot make a complete vacuum environment, which results in residual air, the external oxygen gradually permeates into the packaging under the effect of internal and external pressure differences.

It is also speculated that a high concentration of carbon dioxide not only controls the growth of microorganisms, but also changes the pH on the surface of the fish, thus changing the activity of protease and affecting the degradation of myofibrils.

Unlike the results of Lametsch and Lonergan, which showed that protein oxidation reduces the activity of µ-calpain cysteine [48], thus inhibiting the degradation of muscle fibril in pork, the reason here may be that the low rate of protein oxidation could enhance the sensitivity of proteolytic enzymes for myofibril [14], and the activity of protease loss is not obvious under weak oxidation. Thus, fish and other meat proteasome systems also show large differences, which also might be the reason for the difference between the experimental results. Therefore, further research is needed on the metabolic pathway of how the protein oxidation of grouper muscle affects protein degradation.

## 4. Conclusions

This study shows that the changes of carbonyl, sulfhydryl, and Ca^2+^ ATPase activity greatly varied with prolonged storage time, and demonstrates that the degree of grouper fillet protein oxidation was increased. The amide I band absorption peak of the infrared moved towards a higher wavenumber, while the secondary structure of the α-helix gradually transformed into a random curl. It has been shown that great changes of protein structure took place in grouper fillets during refrigerated storage. By combining indexes of protein degradation, such as MFI, SDS-PAGE, and TCA-soluble peptide content, it is concluded that myofibril oxidation could promote protein degradation in grouper fillets during refrigerated storage, which could be observed in each group (AP, VP, and MAP). High-carbon-dioxide MAP played a positive role in the inhibition of myofibril degradation and oxidation for refrigerated grouper fillets.

## Figures and Tables

**Table 1:** Changes in free amino acid (FAA) content (mg/100 g) of grouper muscle during refrigerated storage at 4 °C.

FAA	Day 0	AP _Day 6_	AP _Day 12_	VP _Day 6_	VP _Day 12_	MAP _Day 6_	MAP _Day 12_
Asp	5.69 ± 0.13 ^a^	1.93 ± 0.12 ^c^	1.09 ± 0.06 ^c^	3.15 ± 0.12 ^b^	1.71 ± 0.12 ^c^	1.49 ± 0.05 ^c^	3.32 ± 0.10 ^b^
Thr	14.24 ± 0.48 ^b^	20.57 ± 1.55 ^a^	13.41 ± 1.10 ^b,c^	20.84 ± 1.42 ^a^	12.71 ± 0.14 ^c^	12.65 ± 0.23 ^c^	13.09 ± 0.15 ^b,c^
Ser	20.24 ± 0.60 ^b^	25.79 ± 0.16 ^a^	13.78 ± 0.25 ^b,c^	21.87 ± 1.57 ^a^	12.25 ± 0.07 ^c^	12.19 ± 0.11 ^c^	14.01 ± 0.12 ^b,c^
Glu	8.71 ± 0.30 ^d^	27.20 ± 0.09 ^a,b^	18.59 ± 0.31 ^c^	18.34 ± 1.3 ^c^	22.06 ± 0.1 ^b,c^	30.98 ± 0.10 ^a^	22.73 ± 0.01 ^b,c^
Gly	91.14 ± 2.98 ^b^	153.15 ± 1.21 ^a^	78.74 ± 0.81 ^c^	114.93 ± 7.94 ^a,b^	88.33 ± 0.1 ^b,c^	84.20 ± 0.85 ^b,c^	108.33 ± 1.75 ^a,b^
Ala	23.54 ± 0.78 ^b,c^	43.80 ± 0.40 ^a^	25.55 ± 0.25 ^b,c^	27.65 ± 1.89 ^b,c^	22.88 ± 0.04 ^b,c^	30.04 ± 0.84 ^b^	28.55 ± 0.49 ^c^
Cys	1.23 ± 0.03 ^a^	0.95 ± 0.11 ^a,b^	0.48 ± 0.07 ^c^	0.78 ± 0.09 ^a,b^	0.24 ± 0.20 ^d^	1.56 ± 0.09 ^a^	0.48 ± 0.05 ^c^
Val	3.69 ± 0.14 ^c^	4.99 ± 0.12 ^b,c^	3.90 ± 0.09 ^c^	5.52 ± 0.38 ^b^	2.91 ± 0.06 ^c^	7.81 ± 0.04 ^a^	5.77 ± 0.12 ^b^
Met	1.70 ± 0.19 ^d^	2.18 ± 0.20 ^c^	1.90 ± 0.13 ^d^	2.87 ± 0.11 ^b,c^	1.16 ± 0.10 ^d^	6.25 ± 0.11 ^a^	3.25 ± 0.08 ^b^
Ile	2.64 ± 0.15 ^c^	3.43 ± 0.03 ^b,c^	2.74 ± 0.11 ^c^	3.89 ± 0.41 ^b^	2.03 ± 0.13 ^c^	5.47 ± 0.82 ^a^	3.85 ± 0.03 ^b^
Leu	4.18 ± 0.03 ^c^	5.54 ± 0.09 ^b^	4.44 ± 0.09 ^c^	6.28 ± 0.49 ^b^	3.12 ± 0.05 ^c^	9.05 ± 0.03 ^a^	6.39 ± 0.12 ^b^
Tyr	1.61 ± 0.28 ^c^	2.64 ± 0.26 ^b,c^	1.55 ± 0.49 ^c^	2.64 ± 0.37 ^b,c^	1.62 ± 0.12 ^c^	6.05 ± 0.85 ^a^	2.93 ± 0.02 ^b,c^
Phe	4.15 ± 0.39 ^d^	5.34 ± 0.21 ^c,d^	5.98 ± 0.14 ^c^	6.57 ± 0.85 ^b,c^	5.91 ± 0.39 ^c^	9.54 ± 0.94 ^a^	7.01 ± 0.42 ^b,c^
Lys	33.66 ± 1.04 ^b^	39.20 ± 0.29 ^a,b^	30.85 ± 0.33 ^c^	38.60 ± 2.87 ^a,b^	36.00 ± 0.20 ^b^	43.79 ± 0.27 ^a^	28.30 ± 0.46 ^c^
His	3.39 ± 0.25 ^c^	5.27 ± 0.18 ^a^	3.09 ± 0.91 ^c^	4.74 ± 0.38 ^b,c^	2.16 ± 0.10 ^c,d^	4.61 ± 0.46 ^b,c^	3.82 ± 0.34 ^b,c^
Arg	4.57 ± 0.12 ^c^	9.38 ± 0.48 ^a^	7.61 ± 1.85 ^a,b^	8.81 ± 0.49 ^a^	5.82 ± 0.36 ^b,c^	7.76 ± 0.37 ^b,c^	5.24 ± 0.14 ^a,b^
Pro	7.55 ± 0.26 ^d^	10.67 ± 0.19 ^b^	8.76 ± 0.15 ^c,d^	17.42 ± 1.30 ^a^	9.28 ± 0.07 ^b,c^	18.78 ± 0.07 ^a^	8.04 ± 0.37 ^c,d^
total	231.91 ± 6.67 ^c^	362.03 ± 4.03 ^a^	219.49 ± 3.25 ^c^	304.89 ± 21.5 ^b^	230.19 ± 0.51 ^c^	292.23 ± 6.03 ^b,c^	265.09 ± 3.92 ^b,c^

Results are expressed as means in mg per 100 g of sample with standard errors. Different lower-case letters (^a^, ^b^, ^c^ and ^d^) in different groups for same amino acid indicate a significant difference (*p* < 0.05). AP: air packaging group; VP: vacuum packaging group; MAP: modified atmosphere packaging group.

**Table 2:** Correlation analysis between protein oxidation and the degradation of grouper in three groups.

Group	Indicator of Protein Oxidation	Indicator of Protein Degradation	Pearson Correlation Coefficient
AP	Carbonyl content	TCA-soluble peptide content	0.683
AP	Total sulfhydryl content	TCA-soluble peptide content	−0.981 **
AP	Carbonyl content	Myofibril fragmentation index	0.956 *
AP	Total sulfhydryl content	Myofibril fragmentation index	−0.961 *
VP	Carbonyl content	TCA-soluble peptide content	0.941 *
VP	Total sulfhydryl content	TCA-soluble peptide content	−0.975 *
VP	Carbonyl content	Myofibril fragmentation index	0.940 *
VP	Total sulfhydryl content	Myofibril fragmentation index	−0.975 **
MAP	Carbonyl content	TCA-soluble peptide content	0.793
MAP	Total sulfhydryl content	TCA-soluble peptide content	−0.982 **
MAP	Carbonyl content	Myofibril fragmentation index	0.934 *
MAP	Total sulfhydryl content	Myofibril fragmentation index	−0.929 *

AP: air packaging; VP: vacuum packaging; MAP: modified atmosphere packaging. * correlation ** strong correlation.

## References

[1] Kirtil E., Kilercioglu M., Oztop M.H. (2016). Modified atmosphere packaging of foods. Reference Module in Food Science.

[2] Guimarães C.F.M., Mársico E.T., Monteiro M.L.G., Lemos M., Mano S.B., Junior C.A.C. (2016). The chemical quality of frozen vietnamese pangasius hypophthalmus fillets. Food Sci. Nutr..

[3] Zhao H., Liu S., Tian C., Yan G., Wang D. (2018). An overview of current status of cold chain in china. Int. J. Refrig..

[4] Estévez M. (2011). Protein carbonyls in meat systems: A review. Meat Sci..

[5] Turgut S.S., Işıkçı F., Soyer A. (2017). Antioxidant activity of pomegranate peel extract on lipid and protein oxidation in beef meatballs during frozen storage. Meat Sci..

[6] Ge L., Xu Y., Xia W., Jiang Q., Jiang X. (2016). Differential role of endogenous cathepsin and microorganism in texture softening of ice-stored grass carp (ctenopharyngodon idella) fillets. J. Sci. Food Agric..

[7] Lu H., Liu X., Zhang Y., Wang H., Luo Y. (2016). Effects of chilling and partial freezing on rigor mortis changes of bighead carp (aristichthys nobilis) fillets: Cathepsin activity, protein degradation and microstructure of myofibrils. J. Food Sci..

[8] Zhang W., Xiao S., Ahn D.U. (2013). Protein oxidation: Basic principles and implications for meat quality. Crit. Rev. Food Sci. Nutr..

[9] Van Haute S., Raes K., Devlieghere F., Sampers I. (2017). Combined use of cinnamon essential oil and map/vacuum packaging to increase the microbial and sensorial shelf life of lean pork and salmon. Food Packag. Shelf Life.

[10] dos Santos P.R., Donado-Pestana C.M., Delgado E.F., Tanaka F.O., Contreras-Castillo C.J. (2015). Tenderness and oxidative stability of nellore bulls steaks packaged under vacuum or modified atmosphere during storage at 2 °C. Food Packag. Shelf Life.

[11] Łopacka J., Półtorak A., Wierzbicka A. (2016). Effect of map, vacuum skin-pack and combined packaging methods on physicochemical properties of beef steaks stored up to 12days. Meat Sci..

[12] Aguilera Barraza F.A., León R.A.Q., Álvarez P.X.L. (2015). Kinetics of protein and textural changes in atlantic salmon under frozen storage. Food Chem..

[13] Pan S., Wu S. (2014). Effect of chitooligosaccharides on the denaturation of weever myofibrillar protein during frozen storage. Int. J. Biol. Macromol..

[14] Yang F., Jing D., Yu D., Xia W., Jiang Q., Xu Y., Yu P. (2019). Differential roles of ice crystal, endogenous proteolytic activities and oxidation in softening of obscure pufferfish (takifugu obscurus) fillets during frozen storage. Food Chem..

[15] Davies K.J.A. (2001). Degradation of oxidized proteins by the 20 s proteasome. Biochimie.

[16] Berardo A., Claeys E., Vossen E., Leroy F., De Smet S. (2015). Protein oxidation affects proteolysis in a meat model system. Meat Sci..

[17] Ogawa M., Nakamura S., Horimoto Y., An H., Tsuchiya T., Nakai S. (1999). Raman spectroscopic study of changes in fish actomyosin during setting. J. Agric. Food Chem..

[18] Abbey L.D., Glover-Amengor M., Atikpo M.O., Howell N.K. (2017). Proximate and biochemical characterization of burrito (bachydeuterus auritus) and flying gurnard (dactylopterus volitans). Food Sci. Nutr..

[19] Oliver C.N., Ahn B.W., Moerman E.J., Goldstein S., Stadtman E.R. (1987). Age-related changes in oxidized proteins. J. Biol. Chem..

[20] Chelh I., Gatellier P., Santé-Lhoutellier V. (2006). Technical note: A simplified procedure for myofibril hydrophobicity determination. Meat Sci..

[21] Benjakul S., Visessanguan W.C., Tanaka M. (2003). Comparative study on physicochemical changes of muscle proteins from some tropical fish during frozen storage. Food Res. Int..

[22] Thannhauser T.W., Konishi Y., Scheraga H.A. (1987). Analysis for disulfide bonds in peptides and proteins. Methods Enzymol..

[23] Benjakul S., Seymour T.A., Morrissey M.T., Haejung A.N. (2010). Physicochemical changes in pacific whiting muscle proteins during iced storage. J. Food Sci..

[24] Thanonkaew A., Benjakul S., Visessanguan W., Decker E.A. (2006). The effect of metal ions on lipid oxidation, colour and physicochemical properties of cuttlefish (sepia pharaonis) subjected to multiple freeze–thaw cycles. Food Chem..

[25] Sriket C., Benjakul S., Visessanguan W., Hara K., Yoshida A. (2012). Retardation of post-mortem changes of freshwater prawn (macrobrachium rosenbergii) stored in ice by legume seed extracts. Food Chem..

[26] Culler R.D., Parrish F.C., Smith G.C., Cross H.R. (2010). Relationship of myofibril fragmentation index to certain chemical, physical and sensory characteristics of bovine longissimus muscle. J. Food Sci..

[27] Yu D., Xu Y., Regenstein J.M., Xia W., Yang F., Jiang Q., Wang B. (2018). The effects of edible chitosan-based coatings on flavor quality of raw grass carp (ctenopharyngodon idellus) fillets during refrigerated storage. Food Chem..

[28] Verrez-Bagnis V., Ladrat C., Morzel M., Noël J., Fleurence J. (2015). Protein changes in post mortem sea bass (dicentrarchus labrax) muscle monitored by one-and two-dimensional gel electrophoresis. Electrophoresis.

[29] Biswas S., Chida A.S., Rahman I. (2006). Redox modifications of protein-thiols: Emerging roles in cell signaling. Biochem. Pharmacol..

[30] Soyer A., Özalp B., Dalmış Ü., Bilgin V. (2010). Effects of freezing temperature and duration of frozen storage on lipid and protein oxidation in chicken meat. Food Chem..

[31] Nikoo M., Benjakul S., Rahmanifarah K. (2016). Hydrolysates from marine sources as cryoprotective substances in seafoods and seafood products. Trends Food Sci. Technol..

[32] Zhang L., Gui P., Zhang Y., Lin J., Li Q., Hong H., Luo Y. (2019). Assessment of structural, textural, and gelation properties of myofibrillar protein of silver carp (hypophthalmichthys molitrix) modified by stunning and oxidative stress. LWT-Food Sci. Technol..

[33] Yarnpakdee S., Benjakul S., Visessanguan W., Kijroongrojana K. (2009). Thermal properties and heat-induced aggregation of natural actomyosin extracted from goatfish (mulloidichthys martinicus) muscle as influenced by iced storage. Food Hydrocoll..

[34] He Y., Huang H., Li L., Yang X., Hao S., Chen S., Deng J. (2018). The effects of modified atmosphere packaging and enzyme inhibitors on protein oxidation of tilapia muscle during iced storage. LWT-Food Sci. Technol..

[35] Ko W.-C., Shi H.-Z., Chang C.-K., Huang Y.-H., Chen Y.-A., Hsieh C.-W. (2016). Effect of adjustable parallel high voltage on biochemical indicators and actomyosin Ca^2+^-atpase from tilapia (*Orechromis niloticus*). LWT-Food Sci. Technol..

[36] Zhang B., Hao G.-J., Cao H.-J., Tang H., Zhang Y.-Y., Deng S.-G. (2018). The cryoprotectant effect of xylooligosaccharides on denaturation of peeled shrimp (litopenaeus vannamei) protein during frozen storage. Food Hydrocoll..

[37] Yu D., Regenstein J.M., Zang J., Xia W., Xu Y., Jiang Q., Yang F. (2018). Inhibitory effects of chitosan-based coatings on endogenous enzyme activities, proteolytic degradation and texture softening of grass carp (ctenopharyngodon idellus) fillets stored at 4 °C. Food Chem..

[38] Rawdkuen S., Jongjareonrak A., Phatcharat S., Benjakul S. (2010). Assessment of protein changes in farmed giant catfish (pangasianodon gigas) muscles during refrigerated storage. Int. J. Food Sci. Technol..

[39] Taylor R.G., Geesink G.H., Thompson V.F., Koohmaraie M., Goll D.E. (1995). Is z-disk degradation responsible for postmortem tenderization?. J. Anim. Sci..

[40] Shi C., Cui J., Na Q., Luo Y., Han L., Hang W. (2017). Effect of ginger extract and vinegar on atp metabolites, imp-related enzyme activity, reducing sugars and phosphorylated sugars in silver carp during postslaughter storage. Int. J. Food Sci. Technol..

[41] Ruiz-Capillas C., Moral A. (2001). Changes in free amino acids during chilled storage of hake (merluccius merluccius L.) in controlled atmospheres and their use as a quality control index. Eur. Food Res. Technol..

[42] Hanne C.B., Achim K., Ulrike B.C., Ragni O., Andersen H.J. (2006). Heat-induced changes in myofibrillar protein structures and myowater of two pork qualities. A combined ft-ir spectroscopy and low-field nmr relaxometry study. J. Agric. Food Chem..

[43] Carton I., Bocker U., Ofstad R. (2009). Monitoring secondary structural changes in salted and smoked salmon muscle myofiber proteins by ft-ir microspectroscopy. J. Agric. Food Chem..

[44] Tu Z.C., Huang T., Wang H., Sha X.M., Shi Y., Huang X.Q., Man Z.Z., Li D.J. (2015). Physico-chemical properties of gelatin from bighead carp (hypophthalmichthys nobilis) scales by ultrasound-assisted extraction. J. Food Sci. Technol..

[45] Xiong Y.L., Blanchard S.P., Tooru O., Yuanyuan M. (2010). Hydroxyl radical and ferryl-generating systems promote gel network formation of myofibrillar protein. J. Food Sci..

[46] Lu H., Zhang L., Li Q., Luo Y. (2017). Comparison of gel properties and biochemical characteristics of myofibrillar protein from bighead carp (aristichthys nobilis) affected by frozen storage and a hydroxyl radical-generation oxidizing system. Food Chem..

[47] Cayuela J.M., Gil M.D., Bañón S., Garrido M.D. (2004). Effect of vacuum and modified atmosphere packaging on the quality of pork loin. Eur. Food Res. Technol..

[48] Lametsch R., Lonergan S., Huff-Lonergan E. (2008). Disulfide bond within mu-calpain active site inhibits activity and autolysis. Biochim. Et Biophys. Acta Proteins Proteom..

